# The Effect of the Higher Education of Women on Wifehood and Motherhood*Read before the Austin District Medical Society, September 28, 1903.

**Published:** 1903-11

**Authors:** J. W. Carhart

**Affiliations:** Austin, Texas


### TEXAS MEDICAL JOURNAL.

ESTABLISHED JULY, 1885.

PUBLISHED MONTHLY.—SUBSCRIPTION $1.00 A YEAR.

VOL. XIX. AUSTIN, NOVEMBER, 1903. No. 5.

### Original Contributions.

For Texas Medical Journal.

### The Effect of the Higher Education of Women on Wifehood and Motherhood.*

BY J. W. CARHART, M. D., AUSTIN, TEXAS.

The question of the wifehood and motherhood of a nation is a questions of ethics, religion, intelligence, commerce; and, in fact, of the life and perpetuity of the nation itself. As the mother moulds the man; and as the hand that rocks the cradle rules the world, so the destiny of all men is involved in the subject we propose to discuss for a short time. It is plainly, then, worthy of the hour, the place and the occasion, and challenges, for the time, our best powers.

In order to a clear and satisfactory view of the subject, we are compelled to do more than look at the surface of things. Mere facts will not alone furnish data for wise conclusions. Psychological conditions and laws must be studied; and the ever-interesting question of the relation of mind and matter must come within our purview.

He who has neither time, disposition or ability to make these explorations will not be profited by our inquiry. Some may listen, *more canino* by the ever-uppermost idea—the sexual. But the sexual sense may be narcotized, for the time being, while we give our views of the effects of the higher education of women on wifehood and motherhood.

*Read before the Austin District Medical Society, September 28, 1903.

This question is now attracting wide attention among thinkers, and is being discussed by men and women of different callings, character and capacities.

The educator is speaking from his standpoint. But his vision is circumscribed, and too limited, and is apt to show the effect of self-interest.

In *The World's Work* for August, Dr. J. M. Taylor, President of Vassar College, writes on "The Education of Women," discussing especially the bearing of the higher education on the life of women and on their attitude towards marriage and the home. He claims that the health of college women improves during the four years of the college course, and he believes there is little or no bearing upon the matter of marriage and the home; nor does he find, after studying the statistics, that coeducation contributes to favor marriage more than the independent college. In short, he argues that there is nothing in the college training of American women to produce abnormal results.

That Professor Taylor is honest goes without saying; and yet his views may have taken too narrow a scope, and may be, and doubtless are, warped by his vocation.

Of the entirely opposite view is Margaret Bisland, who attempts to show that the tendency of American institutions is directly at variance with the natural maternal instinct which is cherished among Asian and European peoples. The results are seen in our diminished families and in the weakening of the marriage tie. We fail or refuse to see the violently reactionary influence upon the race of that tendency of our Occidental civilization which, in withdrawing the women more and more from their home, tends to destroy the true balance of the physical and moral forces between the sexes. The most marked and deleterious effect of American civilization upon women is the false energies and abnormal ambitions it excites in her life. Her endeavor is no longer for the realization and clarification of her sex in its femininity. The education she receives tends to render her either contemptuous of, or indifferent to, her own peculiar forces and their normal expression. For them she not only strives, but is encouraged to substitute an individuality which is purely hybrid and unessential—a grotesque falsetto masculinity.

I ask your forbearance while I quote another author, Professor J. Jessieu, who, writing on *"Le Krach de Intellectualle,"* records the failures of femininism, and of coeducation in particular. He points out that even women's colleges recruit their professional staffs from among men, and claims that women have shown themselves decidedly intellectually inferior. The question of the rights of women has nothing to do with the question of intellectual quality. Legitimate femininism's object is to see that society shall make women suffer as little as possible from their natural inferiority; in other words, shall play the part of a society for prevention of cruelty to animals. But intellectual femininism, as tested in America, says Professor Jessieu, has proved a failure.

After giving, at some length, different views of different authors from different view-points, we have to say that we have opinions of our own which we desire to express; not as to whether women are intellectually inferior or superior to men, nor whether they are capable of maintaining themselves, intellectually and financially, in the professions. Others have given emphatic expression of their views on this subject; as, for example, was done by the trustees of the Woman's Medical College of the Northwestern University, at Evanston, Illinois. Their expressed and emphatic reasons for closing said college was the fact that, in their opinion, women had proved themselves failures in the medical profession.

The education of women may properly be divided into the lighter education and the higher education.

Under the lighter education may be considered music, painting, drawing, needlework, botany, rhetoric, common English, and, perhaps, modern languages.

The higher education would include the higher mathematics, Latin, Greek, and one or two of the modern languages, such as French, Spanish or German, physiology, physics, chemistry, geology, history, philosophy, psychology, biology, astronomy, with possibly several other branches. In short, it embraces the idea of a regular college course, the same as for men.

Such an educational course contemplates a preparatory course of, say, two years, followed by a college course of four years, of close, consecutive, hard mental application, under constant strain of competition and examinations.

The question is not one of coeducation, or independent education. It has long been our opinion, from experience and observation as a teacher, both in schools of coeducation of the sexes and in at least one female college, that, while the plan of coeducation develops a more normal sexual life, the separate education of women develops an abnormal sexual life, overstimulating by the excitement of the imagination, or that lack of self-control engendered by the presence and society of the male sex.

It is a fact, seemingly not generally known, that the study of music, poetry and the fine arts, such as painting, sculpture, landscape gardening and needlework, develops the sexual life, or passions, as does rhetoric and oratory; whilst the study of the exact sciences, particularly the higher mathematics, such as algebra, geometry, conic sections, trigonometry, surveying, navigation and astronomy, has the opposite effect.

Close, hard, long-continued study dwarfs the sexual life. Many college professors have few or no children. Impotence with this class is not uncommon. This has become so noticeable as to attract the attention of leading educators.

When at the New York Polyclinic, many years ago, the late Paul F. Mondé told me that he had nineteen female school teachers under his professional treatment, nearly all of whom were suffering from infantile uteri.

My own observation as a practitioner of medicine for a quarter of a century, in which I have paid considerable attention to the treatment of diseases peculiar to women, has shown that many, if not most, literary and educated women coming under my care were suffering from some form of retarded development of the reproductive organs. Thousands of such women marry, from one consideration or another, while many more thousands do not.

There is in comparatively few of these the real "homing instinct." Many of them may, in a manner, be good housekeepers, but lack the native instinct to cultivate, develop and maintain the home life, so essential to true happiness and the development of a great nation and people.

There is a manifest aversion to the production of children, even when there is a full capacity, and as a result there is a lack of the reproduction of the better classes, a condition which our strenuous President has fittingly denominated "race suicide."

The facetious remark that, in Boston, the self-assumed seat of culture, children are born with two-story heads with mansard roofs on, is scarcely worth our notice, except as an illustration of the fact that, as human beings rise in the scale of intelligence, the infant brain and cranial developments are greater, thus enhancing the sufferings of the mother at parturition, and increasing the dangers attending maternity. It is, therefore, not surprising that women of the higher education shrink from the ordeal.

The Indian woman, feeling the pangs of parturition, steps out of the line of march into some favoring shelter, gives birth to her baby, which she picks up in her apron, trudging on, with accelerated step, to overtake the procession.

The *rationale* of genito-urinary conditions consequent upon undeveloped uteri in women of severe mental application in the study of the branches of a college education is, to my mind, easy of explanation.

The physical law of the tendency of blood to those parts of the human economy subject to greatest activity is well understood. In intense application of the mind, the blood tends to the brain, which is thereby nourished and strengthened for its proper functions. After a hearty meal the blood tends to the stomach for its nourishment, that it may properly functionate. The woman who applies herself to the mastery of the higher branches of knowledge, more particularly the mathematics, must, for considerable periods, follow lines of close, consecutive thought, diverting thought and blood from the sexual organs, thereby interfering with the nutrient supply essential to the full and complete development of the reproductive organs. This condition, in time, becomes a habit, and the usual unpleasant consequences follow. The condition of anæmia or of hyperæmia is prejudicial to health and the right function of a given part.

The woman of wealth, luxury, ease, pleasure, self-indulgence and mental lassitude, by the very force of abnormal conditions, dwells on sexual subjects, thereby stimulating her sexual life to the high-water mark of absolute intolerance, which Count Tolstoi has so vividly depicted in his "Kreutzer Sonata."

This protracted sexual hyperæmia is as destructive of health and morals as the opposite is of healthy wifely and motherly instincts.

I would not, on any consideration, place myself on record as opposed to the education of women. I would not take the ground, at present, that they are intellectually inferior to men, although science has demonstrated that the average female brain weighs five ounces less than the average male brain. The average weight of the male brain is forty-nine ounces, and the average weight of the female brain forty-four ounces. The heaviest normal male brain weighed sixty-five ounces, and the heaviest normal female brain weighed fifty-six ounces.

These demonstrated facts must be taken into consideration by those who discuss the question of comparative mental ability, although they may not be absolutely conclusive in themselves.

I am not opposed to women enjoying all the rights calculated to promote their best interests and the interests of the race. But I do maintain that, whilst there are exceptionally endowed women, like Mrs. Browning, George Eliot, Phœbe and Alice Cary, and Miss

Francis E. Willard, who ought to develop their mental powers to their utmost, for the good of humanity, even at the sacrifice of the wifely and motherly instinct, we believe that we are pressing the matter of the college education of women, along the usual lines prescribed for men, at the cost of those more lovely, beautiful, wifely and motherly "homing instincts" which are the crowning glory of womanhood, and which are becoming more and more valuable as women rise to their essential dignity and grandeur as a factor in developing the American nation to God's idea of a great and typical republic.

The rose may be developed to such queenly perfection as to lose its reproductive powers; the orange may be cultivated to such golden fruitage as to become seedless; the strawberry, than which God might have made a better, but never did, may reach a perfection which carries it beyond the point of fecundation.

What is true in the vegetable world is true also among the human species. Man has never reached a point beyond which he could not develop. A late French writer holds that animals are superior to man, because they perfectly answer the end of their being, whilst man does not. How far man may be developed, mentally and physically, is impossible to tell. The growing of men and women is an art and a science, and must be pursued along rational lines.

We now have schools of every description, except for the training of women for wives and mothers. Such schools will be established, and will cover a wide field in their curriculum. They will teach rational methods of attracting the opposite sex—the relation of lovers, and ethics governing them; the relation of husband and wife, their liberties and limitations; the power and influence of woman, as lover, wife and mother; the relations of the mother to the State, church and all eleemosynary institutions and enterprises; her relation to son, daughter and servant, together with instructions in hygiene of person, home and family. Also the moral and intellectual education of husband and children, and the care and nursing of the sick.

This is not as Utopian as might at first sight be supposed; nor is it altogether original, for much of this was taught, in a modified form, by the Athenians, who developed a manhood and womanhood beside which ours seems inferior.

This is a broad subject, which educated physicians can not afford to relegate to quacks, nor even to the teachers of science and general learning.

